# Uranium oxides structural transformation in human body liquids

**DOI:** 10.1038/s41598-023-31059-z

**Published:** 2023-03-11

**Authors:** Tatiana Poliakova, Anna Krot, Alexander Trigub, Iurii Nevolin, Alexey Averin, Vasiliy Yapaskurt, Irina Vlasova, Petr Matveev, Stepan Kalmykov

**Affiliations:** 1 Chemistry Department, Lomonosov Moscow State University, Moscow, 119991 Russian Federation; 2 National Research Center “Kurchatov Institute”, Akademika Kurchatova pl. 1, 123182 Moscow, Russia; 3 Russian Academy of Sciences, Frumkin Institute of Physical Chemistry, Moscow, 119072 Russian Federation; 4 Geology Department, Lomonosov Moscow State University, Moscow, 119991 Russian Federation

**Keywords:** Chemistry, Inorganic chemistry, Nuclear chemistry

## Abstract

Uranium oxide microparticles ingestion is one of the potential sources of internal radiation doses to the humans at accidental or undesirable releases of radioactive materials. It is important to predict the obtained dose and possible biological effect of these microparticles by studying uranium oxides transformations in case of their ingestion or inhalation. Using a combination of methods, a complex examination of structural changes of uranium oxides in the range from UO_2_ to U_4_O_9_, U_3_O_8_ and UO_3_ as well as before and after exposure of uranium oxides in simulated biological fluids: gastro-intestinal and lung—was carried out. Oxides were thoroughly characterized by Raman and XAFS spectroscopy. It was determined that the duration of expose has more influence on all oxides transformations. The greatest changes occurred in U_4_O_9_, that transformed into U_4_O_9-y._ UO_2.05_ and U_3_O_8_ structures became more ordered and UO_3_ did not undergo significant transformation.

## Introduction

Uranium oxides could be spread into the environment as a result of various accidental and undesirable scenarios, such as various operations during uranium mining, accidents at nuclear fuel cycle facilities, wind erosion of contaminated soils, etc^1–4^. Micrometer and submicrometer sized oxide particles could be easily ingested in the immediate vicinity of the contaminated area by both humans and animals^5,6^. Understanding the resistance of uranium oxides towards degradation and dissolution in biological liquids is an important task for predicting the dose effect and toxicity^2,3,5–9^. The understanding of the biotransformation processes in vivo is also important for further treatment detoxing protocols.

The oxidation of uranium dioxide (UO_2_) and the formation of series of hyperstoichiometric oxides UO_2+x_ has been extensively studied in application for nuclear fuel technology and SNF management^10–13^. The further incorporation of oxygen at octahedral interstitials leads to the formation of mixed-valence oxide U_4_O_9_ and U_3_O_8_ and UO_3_ as a result of uranium sublattice changes as well as oxygen saturation^11,14–18^. The dissolution and destruction kinetics and products depend on the uranium oxidation state.

Changes in the composition and properties of microparticles under well-defined laboratory conditions could be examined thoroughly including determination of uranium redox speciation by spectrometric methods such as XANES^11,14,15,19,20^, determination of the local molecular environment of uranium in oxides by EXAFS, structural changes in uranium^10,11,13^ and oxygen^12,18,21^ sublattice by X-ray and neutron diffraction and Raman spectroscopy^13,16,17,22–27^. X-ray absorption near-edge structure (XANES) spectroscopy and extended X-ray absorption fine structure (EXAFS) spectroscopy can provide the information on the oxidation state and local environment of atoms, respectively. Both these methods cover the full X-ray absorption spectra and can be used for clarification of local structure changes for phase comparison.

The solubility of micro- and submicroparticles in various liquids of biological significance has been studied widely. In vitro studies indicative the small difference in dissolved fraction percent between simulated lung fluids like Gamble's and Ringer's solutions and serum ultrafiltrate simulants^5–7^. At a long soaking time the percentage of undissolved fraction of uranium dioxide particles in Gamble’s solution is between 98 and 100%, which is larger than in Ringer’s solution by one to two percent, which is not significant for the particles of the same size. It was found that UO_2_ nanoparticles inhaled by mice remained in the lung with only 1/5 fraction was dissolved and transferred into body fluids with half-life of 2.4 h, while 4/5 of particles had lung retention half-life of 141.5 days for uranium dioxide nanoparticles^9^. For UO_3_ the authors found out the difference between in vivo and in vitro experiments, and for UO_2_ and U_3_O_8_ the results in both experimental modes were consistent^28^. Radiographic studies showed that environmental uranium dioxide microparticles size directly influenced the soluble fraction in simulated lung fluids—with an increase in diameter from 1 to 60 μm^5^.

It is important that micro- and submicroparticles dissolution kinetics strongly depends on the uranium oxidation state as have been shown for environmental samples: non- or low-oxidized, dioxide, particles are more kinetically stable than oxidized up to U_3_O_8_^29^. However, the dependence of uranium oxides behavior in human body liquids on the oxidation state is still an open question. In the above works the change in the composition of the liquid, the number of particles, and the radioactivity of the samples before and after the solubility experiment was studied while subtle changes in the oxidation state and local environment after soaking in biologically significant liquids have not been investigated.

This work is devoted to track the changes in the properties and composition of synthesized uranium oxides with different uranium oxidation states in vitro as a result of their expose into simulated biological liquids: lung, gastric and intestinal ones in order to predict the personal dose rate in case of ingestion or inhalation of a microparticle of uranium oxide, depending on oxidation states of uranium.

## Experiment

### Samples synthesis

Samples of uranium oxides with various degree of oxidation were synthetized in the following way. The precursor was UO_2.05_, a powder of depleted uranium dioxide, by-product of fuel enrichment (JSC ELEMASH Machine-Building Plant, Elektrostal, Russia), partially oxidized due to long-term storage in air. Stoichiometric UO_2_ was obtained by reductive annealing UO_2.05_ at 1900 °C.

The oxide UO_2+x_, with x = 0.10; 0.15 and 0.20, were synthetized from UO_2.05_ powder by using NETZSCH STA 449 F3 Jupiter thermoanalytical complex in synthetic air atmosphere at 130 °C according to the method described by Leinders et al.^10^. It was kept at a certain temperature until the sample mass increase reach the corresponding O/M value.

For β-U_4_O_9_ synthesis heating of an equimolar mixture of crushed ceramic UO_2_ and U_3_O_8_, obtained by decomposition of hexahydrate uranyl nitrate, was held. Quartz ampoule with oxides mixture was evacuated, sealed and heated to a temperature of 1050 °C^30^.

U_3_O_8_ was synthetized by decomposition of hexahydrate uranyl nitrate at 625 °C.

For α-UO_3_ synthesis the method described by Cordfunke was used^31^ e.g. thermal decomposition at a temperature of 525 °C of unwashed uranyl peroxide, which was synthetized by reacting a solution of uranyl nitrate with hydrogen peroxide.

All synthesized samples were characterized by powder X-ray diffraction (Appendix A1), and for β-U_4_O_9_ neutron diffraction was used and the lattice parameters were refined (Appendix A2, A3).

**Figure 1:**
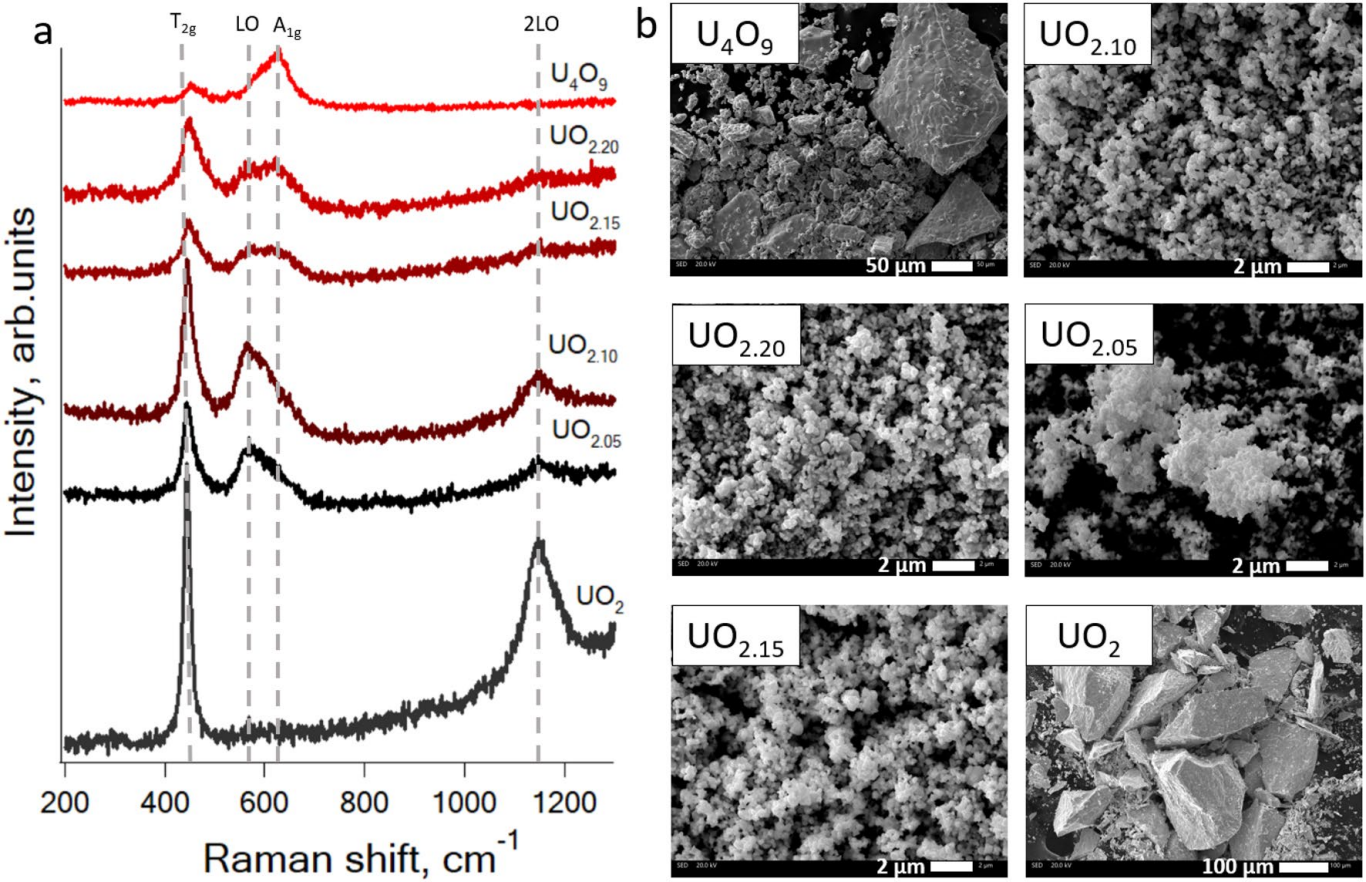
(**a**) Raman spectra of uranium oxides from UO_2_ to U_4_O_9_. (**b**) SEM images of uranium oxides from UO_2_ to U_4_O_9._

The size of the synthetized oxide particles varies from 100 nm to the first tens of microns, which is fall in the range of inhalable particles (Fig. 1b).

### Liquid composition and dissolution experiment

The composition of simulated of human body liquids is shown in Table 1.

**Table 1:** Simulated human body fluids composition.

Stomach^32^		Intestine^32^		Lungs (Gamble solution)^33^	
Reagent	Concentration, g l^−1^	Reagent	Concentration, g l^−1^	Reagent	Concentration, g l^−1^
CaCO_3_	0.2	CaCO_3_	0.2	NaCl	6.79
MgCO_3_	0.2	MgCO_3_	0.2	NaH_2_PO_4_	0.24
KCl	0.67	KCl	0.67	NaHCO_3_	2.27
NaCl	2.8	NaCl	2.8	NH_4_Cl	0.53
Sodium lactate	0.25	Sodium lactate	0.25	CaCl_2_	0.02
Citric acid	0.4	Citric acid	0.4	Glycine	0.45
Carbamide	0.3	Carbamide	0.3	l-cysteine	0.12
Pepsin	1	Pepsin	1	Sodium citrate	0.05
		Glucose	0.4		
		Pancreatin	2		
pH	2 (HCl)	pH	7 (NaOH)	pH	7.4

Oxide sample (5 mg) in 1.5 ml plastic test tube was mixed with 1 ml of simulated human body liquid. The experiment was done in static conditions at 37 °C. The exposure time in stomach liquid was 2 h, after that the liquid was removed, and 1 ml of intestine liquid was added. The stomach liquid after the exposure was mechanically removed after centrifuge. Fresh stomach liquid was modified by adding pancreatin, glucose and NaOH up to reaching pH 7. The resulting intestine liquid was added and the pH was controlled after adding to the sample. The time of expose in intestine liquid was 4 h.

The time of expose in lung fluid at the same temperature was 34 days. According to in vitro studies of the solubility of various uranium oxides under static conditions, the experiment time in lung fluids varied from 20 to 60 days^34–36^. And according to in vivo studies^9^, there are two types of uranium dioxide nanoparticles behavior in lungs: 20% of them have a half-life of 2.4 days, and 80%—141.5 days. Therefore, it was decided to take the value of the time of the experiment averaged according to the literature data—34 days. pH was controlled during the whole experiment.

After the experiment the liquid was removed mechanically by centrifuge, and the particles were air-dried at a room temperature. Cellulose was added to the sample of uranium oxide and pressed pellets were prepared for XANES and EXAFS analyses. The mass of U in the pellet was calculated using HEPHAESTUS software^37^.

### Pre- and post- exposure samples characterization

Phase composition of synthetized oxides was determined by powder X-ray diffractometer Panalytical Aeris with CuKα tube radiation and PIXel3D area detector. Diffraction patterns were recorded in Bragg–Brentano configuration with a step size of 0.011°. Sample powder was placed on zero-background Si sample holder and registered at room temperature. Neutron diffraction was held with high resolution Fourier-diffractometer at research neutron reactor IBR-2 at a room temperature in the Joint Institute for Nuclear Research, Dubna, Russia. The oxides morphology before and after the dissolution experiment was established by scanning electron microscope Jeol JSM-6480LV with INCA Energy-350 at a room temperature in back-scattering and secondary electrons modes. Raman spectra were obtained with a Renishaw inVia Reflex Microscope system equipped with a Peltier-cooled CCD. The 633‐nm lines of a He–Ne laser was used for excitation. Laser light was focused on the sample through a 50× objective to a spot size of ~ 2 μm. The power on the sample was < 0.1 mW. Experimental spectra were decomposed into several components using Fityk software^38^.

U L3 XANES and EXAFS spectra for oxidation state and uranium local molecular environment determination were held of following facilities. Spectra on the initial oxides were obtained on synchrotron radiation source KISI, NRC «Kurchatov Institute», Moscow, Russia, station STM^39^, with monochromator Si (220) and Amptek detector.

U L3 XANES and EXAFS spectra of the oxides after the exposure were obtained on the Rossendorf beamline (ROBL/BM20^40^) of European synchrotron radiation facility ESRF, Grenoble, France (experiment number A20-1-836) with 18-element germanium detector, Ar-N_2_ gas mixture and Si (111) monochromator.

For data evaluation the IFFEFIT package was used^37^. The analysis of raw XAS data was performed in the ATHENA software. Each scan was deglitched and aligned and several scans (2–3) were merged to improve signal-to-noise ratio. Merged spectra were treated carefully to set the correct pre-edge and post-edge lines, E_0_ position. EXAFS fitting was performed in ARTEMIS in R-space, Fourier transform range was 3–13 Å^−1^. Structural information on UO_2_, U_3_O_8_ and UO_3_ required for FEFF calculation (for lattice images see Appendix A4) was taken from literature data^41,42^. U_4_O_9_ sample was fitted according to the structure obtained for this sample from neutron diffraction study. Scattering paths required for fitting of EXAFS spectra were calculated using FEFF6 and FEFF8.5 code^43^. During the fitting, S_0_^2^ was set to 0.9, ∆E_0_ was the same for all coordination spheres and varied as a global parameter. Generally, coordination numbers (CNs) were fixed according to crystallographic values. In some samples CNs were allowed to vary for O coordination shell with a limitation of the sum over split subshells. The samples where it was done as well as the reasons are specified in the text. The criteria of acceptance for the fitting were statistical parameters and physical feasibility of the obtained model.

## Results and discussion

The dissolution experiments were carried out for following oxides: UO_2.05_, U_4_O_9_, U_3_O_8_, UO_3_ due to their noticeable difference in their Raman, XANES and EXAFS spectra. The structure of the synthesized oxides UO_2+x_ series was also studied.

### Raman spectroscopy and scanning electron microscopy (SEM)

**Figure 2:**
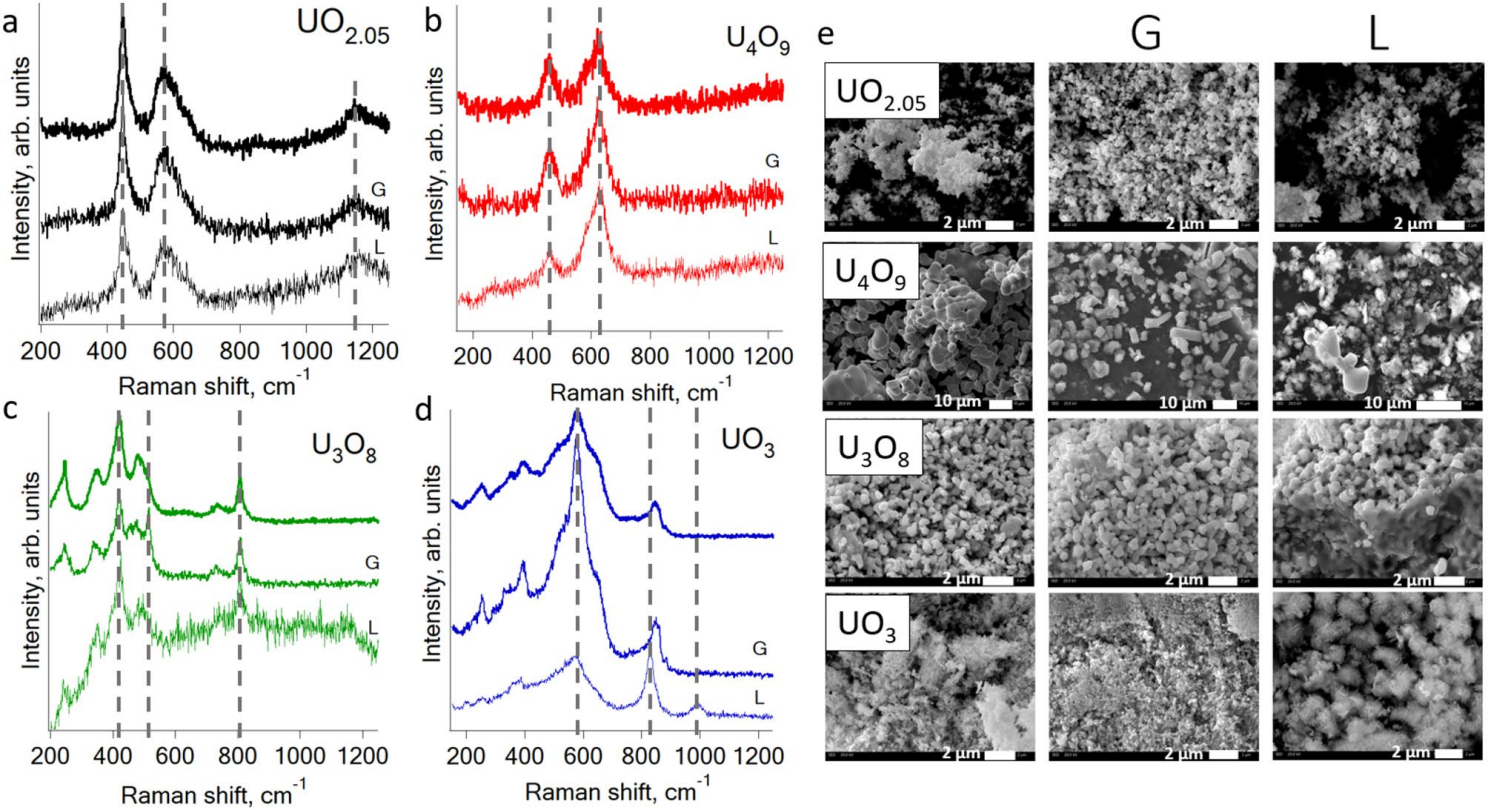
Raman spectra of uranium oxides before and after exposure in simulated lungs (L) and gastro-intestinal (G) liquids: (**a**) UO_2.05_; (**b**) U_4_O_9_; (**c**) U_3_O_8_; (**d**) UO_3_. (**e**) SEM images of UO_2.05_ before and after dissolution.

Raman spectra of stoichiometric UO_2_, hyperstoichiometric UO_2+x_ oxides and U_4_O_9_ along with SEM images are shown in Fig. 1. Raman spectra of UO_2_, U_4_O_9_, U_3_O_8_ and UO_3_ before and after dissolution experiment along with SEM images before and after dissolution are shown in Fig. 2.

### Raman spectroscopy for characterization of synthesized uranium oxides

The Raman spectrum (see Fig. 1a) of stoichiometric uranium dioxide shows peaks at 445 and 1150 cm^−1^ corresponding to the triple degenerate Raman active vibration U–O T_2g_ and the 2LO peak, which is devoted to Γ5–Γ3 crystal field electronic transition^44^. That proves its stoichiometry^22,23,26,45–47^. For hyperstoichiometric oxides, the appearance of the peak at 560 cm^−1^ was observed, which corresponds to a first-order longitudinal optical phonon LO starting from UO_2.05_. With further saturation of the oxygen sublattice, a peak at 630 cm^−1^ is observed, corresponding to the stretching vibration U–O A_1g_. Its appearance is due to the distortion of the anionic sublattice in the fluorite structure as a result of incorporation of oxygen^27,48^. In this case, for UO_2.10_ oxide, the appearance of the second peak looks like a broadening of the LO line. For UO_2.15_ the LO and A_1g_ lines have the same intensity, while for UO_2.20_ the intensity of the second peak becomes higher. This is in a good agreement with the peaks deconvolution results (Table 2, Appendix A4.1). Only the lines corresponding to the T_2g_ and A_1g_ vibrations remain in the spectrum of U_4_O_9_. Raman shift presence in uranium oxides spectra is shown in Table 2.

**Table 2:** Uranium oxides Raman shift peaks characteristics.

Oxide	445 cm^−1^/T_2g_	560 cm^−1^/LO		620 cm^−1^/A_1g_		1050 cm^−1^/2LO
		Peaks deconvolution parameters				
		FWHM	Height	FWHM	Height	
UO_2_	+*	−		−	−	+
UO_2.05_	+	+		−	−	+
UO_2.10_	+	41.9	421.0	10.7	50.4	+
UO_2.15_	+	43.3	146.0	25.2	80.3	−
UO_2.20_	+	41.5	108.2	23.6	121.6	−
U_4_O_9_	+	−	−	+		−

The Raman spectrum (for Raman spectra of UO_2.05_, U_4_O_9_, U_3_O_8_ and UO_3_ see Appendix A5) of U_3_O_8_ contains lines corresponding to the vibrational modes B_1g_, B_2g_, B_3g_, and A_1g_ in the region from 100 to 500 cm^−149^. The peak in the region of 800 cm^−1^ corresponds to vertical vibrations of the uranyl cation or impurity uranium trioxide^26,45,46^. The Raman spectrum of uranium trioxide contains vibrational modes B_1g_, B_2g_, B_3g_ and peaks in regions 590 cm^−1^ and 852 cm^−1^, which is in good agreement with the results of previous studies of α-UO_3_^17^.

Raman spectroscopy enables to detect changes in the crystal lattice, which is shown by a smooth transition from the 560 cm^−1^ to the 630 cm^−1^ peaks in the UO_2_ to U_4_O_9_ range and the appearance of the new scattering lines in the spectra of oxides in higher oxidation states (see Appendix A8).

### Comparison of uranium oxides Raman spectra before and after dissolution experiment

The average particle size for the initial sample UO_2.05_ was about 3 μm, as determined by dynamic light scattering method, which means that the size of particle aggregates was determined, since according to the SEM the particle size is much smaller—around 500 nm. The values of the specific free surface established by processing the complete isotherms of nitrogen sorption at 77 K in the framework of the BET model was rather low—about 3 m^2^ g^−1^.

The particle size and the morphology changes differently for the studied oxides (see Fig. 2e for SEM images). Morphology of UO_2.05_ changes insignificantly, while the average size changed from 500 to 300 nm. U_4_O_9_ particles morphology changes greatly—e.g. after gastrointestinal liquids particles become sharper, and after lung fluid it become flake-like. The average particle size decreases from 7 to 2 μm. For U_3_O_8_ average particle size changes insignificantly as do the morphology. UO_3_ morphology changes after the expose to lung fluid—it become needle-like. The average particle size changes insignificantly.

The spectra (see Fig. 2a and Appendix A7) of UO_2.05_ show the difference in 445 cm^−1^ and 560 cm^−1^ peaks intensity after exposure to both liquids. While the 1050 cm^−1^ peak has approximately the same intensity, the 560 cm^−1^ peak increases relative to 445 cm^−1^ after exposure indicative the increased degree of non-stoichiometry in the fluorite lattice due to a decrease in the contribution of T_2g_ vibration. The peaks maxima have the same positions indicative the stability of the crystal lattice and consequently the resistance of the UO_2.05_ towards dissolution in body fluids. The U_4_O_9_ spectra (see Fig. 2a and Appendix A7) before and after the exposure to liquids of the gastrointestinal tract shows the peak increase at 620 cm^−1^ relative to 445 cm^−1^, which indicates a rising degree of non-stoichiometry in the lattice as it was shown for UO_2.05_ oxide.

The spectra (see Fig. 2a for Raman spectra, Appendix A6 for deconvolution results, A7 for residual of spectra) of U_3_O_8_ become noisy after the exposition in liquids, especially in lungs liquids. The 454 cm^−1^ mode, which is not detected in the initial and lung spectra, become a separate peak after the gastrointestinal tract liquid, according to deconvolution results. The Raman spectrum after the exposure to lung fluid is also noisier than of initial U_3_O_8_. After the exposure to the gastrointestinal tract liquids, a noticeable line appears in the spectrum of U_3_O_8_ in the 500 cm^−1^ region, which may correspond to the first-order longitudinal optical phonon LO, which also appears in hyperstoichiometric UO_2+x_ oxides together with defects in the fluorite structure or a threefold degenerate T_2g_ vibration, which also indicative the appearance of the UO_2+x_ phase*.* The positions of the lines (see Fig. 2a and Appendix A7) for UO_3_ after dissolution also changes significantly. After exposure to gastric fluid, the peak in the 400 cm^−1^ region becomes narrower, which corresponds to E_g_ stretching in the P4_2_/nmc lattice due to the formation of cuboctahedral Willis clusters^25^. After exposure to the lung fluid, peaks at 818 cm^−1^ appear, instead of the peak at 852 cm^−1^ in the initial oxide and after gastrointestinal fluids, and 990 cm^−1^, which may correspond to the peaks of organic impurities^50^. Because of the noise appearing in the spectra after pulmonary fluids due to the presence of organic compounds, the correct interpretation of the spectra is difficult, since low-intensity modes will be leveled by the signal from organic impurities.

Raman spectroscopy shows the greatest susceptibility to changes in the oxygen sublattice in uranium oxides, which is consistent with the results of previous studies^17,22,25^. The stability of UO_2.05_ in all media contradicts with previous studies, which showed that as a result of stoichiometric uranium dioxide soaking, a UO_2.25_ phase is formed on the surface^51,52^. This contradiction could be explained by the fact that in our work, the flow-through reactor was used in the experiment and pH of all the liquids was lower than it was introduced in Torrero et al.^52^. Then the kinetics has more influence in uranium dioxide surface oxidation. Ulrich et al. established the appearance of a passivating UO_2+x_ layer on the surface of the granules under oxidizing conditions. Leinders et al.^10^ showed the formation of disordered U_4_O_9_ at the beginning of the oxidation process of UO_2.03_ granules, after which ordering and further oxidation occurred to form inclusions of amorphous uranium trioxide on the surface. As Raman spectroscopy is sensitive towards oxidative processes from the very beginning in the case of hyperstoichiometric uranium dioxide, the absence of changes in the Raman spectra indicates the absence of oxidation processes on the particles surface. No surface changes in U_4_O_9_, according to Raman spectroscopy are observed, though the lattice disorder degree increases. For U_3_O_8_ we noticed the presence of impurities in a more reduced form of the oxide on the granules surface (peak in the region around 520 cm^−1^).

According to the results of Raman spectroscopy, exposure to lung fluid had the greatest effect on all oxides. This could be explained by the fact that the duration of the experiment with lung fluid was much longer: 34 days compared to 2 + 4 h in gastrointestinal fluids, which is consistent with the previous studies of uranium ore dissolution in simulated lung fluids, where the dissolution equilibrium was reached after 72–120 h^53^. Considering the pH of simulated liquids, we should have expected the greater changes after gastrointestinal liquids then after lung fluids. Above pH 7 the solubility of uranium dioxide decreases more than 3 orders of magnitude^52^. Here we can conclude that the duration of the dissolution experiment has more significant influence than pH on the changes of oxide surface properties.

### XANES and EXAFS

**Figure 3:**
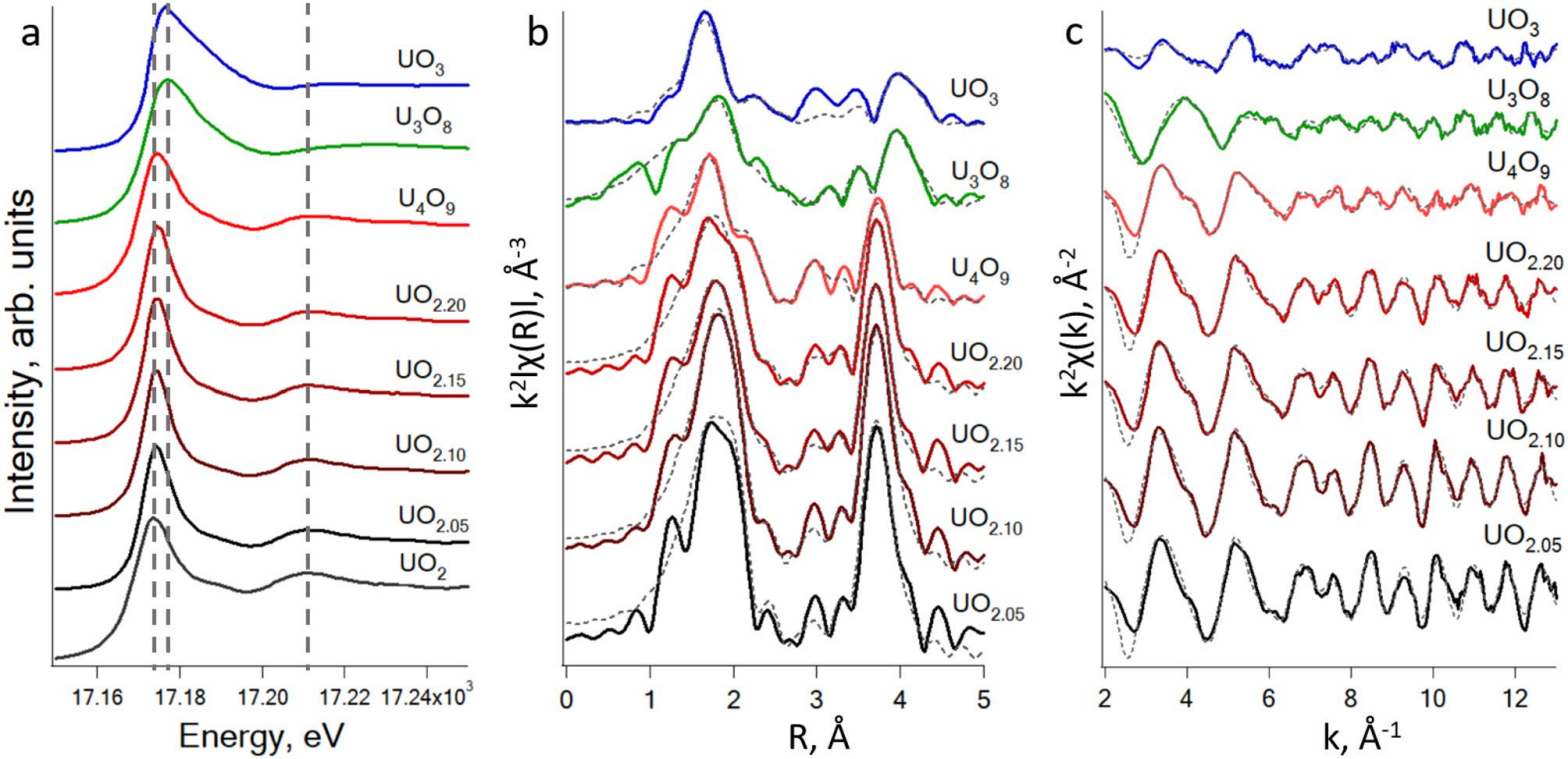
(**a**) XANES spectra at the U L3-edge for uranium oxides in various oxidation states. (**b**, **c**) EXAFS spectra at the U L3-edge for uranium oxides in various oxidation states and their Fourier transforms. Solid lines are for experimental data, dotted lines are for fits.

**Figure 4:**
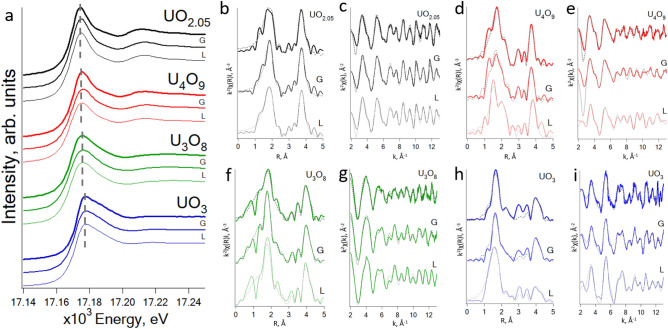
Comparison of U L-3 edge XAS data for initial and exposed to lung (L), gastric and intestinal (G) simulated fluids uranium oxides: (**a**)—XANES; (**b**, **d**, **f**, **h**)—EXAFS spectra; (**c**, **e**, **g**, **i**)—corresponding Fourier transforms of the EXAFS spectra.

To determine the oxidation state of uranium and its local molecular environment in oxides, X-ray absorption spectroscopy (XAS) measurements were performed before and after the solubility experiment. XANES and EXAFS spectra along with fitting curves are shown in Figs. 3 and 4.

### XANES and EXAFS for characterization of synthetized uranium oxides

The position of the white line of the spectra of uranium oxides from UO_2_ to U_4_O_9_ noticeably shifts (by 1–2 eV) towards higher energy when the sublattice is saturated with oxygen within 5 eV, which is consistent with the results of previous studies^14,15,54–59^. The post-edge structures of these spectra have a similar shape with a local maximum at 17,209 eV. Upon oxidation to U_3_O_8_, the energy position of the white line changes more dramatically—i.e. by 10 eV compared to UO_2_. In this case, the peak shape is the same, and the first post-edge feature is in the region of 17,230 eV, which is in the higher energy region compared to oxides with a lower oxidation state of uranium. The position of the white line in the spectrum of uranium trioxide remains the same as in the spectrum of U_3_O_8_, however, the shape of the main absorption peak is less symmetrical: the right edge is flatter, so the peak has the shape of a triangle. Post-edge structures are not observed in the spectrum of uranium trioxide (Fig. 3a).

As additional oxygen atoms are incorporated into the interstitial positions of fluorite type crystal lattice of the UO_2.0_ resulting in the formation of non-stoichiometric series UO_2+x_, the coordination number (CN) of O atoms of the first coordination sphere increases. To take into account the increase of CN due to the incorporation of additional O in the structure, a linear dependence of the CN as a function of the degree of oxide of oxidation, x, is introduced in the fitting models of UO_2+x_. The reference points for the linear extrapolation are the stoichiometric oxides UO_2.0_ (CN = 8) and U_4_O_9_ (CN = 11), as their structure and CN are well known from diffraction data.

According to EXAFS (Appendix A9), UO_2.05_ stands out of the entire series of oxides in terms of the first coordination sphere parameters. In the Fourier transform magnitude of the spectrum, a peak with double maximum is clearly observed at a distance of 1.4–2.2 Å, which corresponds to split O coordination sphere with distances of 2.24 and 2.39 Å. The inclusion of a small amount of additional oxygen causes the displacement of O atoms from regular positions of fluorite-type lattice (2.34 Å) both up and down. In this case, the shift to the smaller distances is two times higher (~ 0.1 Å) then to the larger side (0.05 Å). On the contrary, in oxides with x = 0.10–0.20, the local surrounding remains roughly unchanged and is more consistent with the concept of including additional atoms in the fluorite structure. For these oxides, the fluorite oxygen sublattice is retained at a distance characteristic for UO_2_ with CN of about 8. An additional subshell appears at a ~ 2.8 Å corresponding to O atoms in defect positions. It should be noted that the Debye–Waller parameter increases with the increase of oxides defectiveness, which is a natural consequence of structure disordering as the number of defects increases. The distance to U coordination sphere is the same for all oxides of the series and coincides with the value known for the stoichiometric UO_2_ (3.87 ± 0.01 Å) from earlier EXAFS calculations^60^. A shift of 0.01 Å appears only in UO_2.20_. However, its significance is doubtful due to the uncertainty of interatomic distances calculation from EXAFS being ± 0.01–0.02 Å. Based on the values of the Debye–Waller factors, disorder in the uranium sublattice increases significantly when x = 0.15 is reached, which is expressed by dramatic increase of σ^2^.

Fitting the EXAFS spectra of the series of non-stoichiometric oxides UO_2+x_ with x ranging from 0.05 to 0.20 and comparing the parameters of the local environment with stoichiometric UO_2_ showed the following regularities (see Fig. 3b, c for EXAFS experimental and fit data and Appendix A9 for fitting parameters):

- Small degree of oxidation (x = 0.05) causes distortion of the initial fluorite structure and splitting of the oxygen sublattice into two with approximately equal CNs at distances 2.24 and 2.39 Å, while the uranium sublattice remains unchanged.
- In oxides with high oxidation degree (x = 0.10–0.20), the fluorite oxygen sublattice is stable and additional oxygen atoms appear in interstitial positions at a distance of ~ 2.8 Å from U. The Debye–Waller parameter of O subshells increases with the defectiveness, which correlates with the increasing distortion.
- The uranium sublattice remains unchanged at a distance of 3.87 ± 0.01 Å, consistent to stoichiometric UO_2_. Significant disordering of this sublattice occurs at x = 0.15, which is expressed by a significant increase in the Debye–Waller parameter for UO_2.15_ and UO_2.20_.

XANES makes it possible to demonstrate small changes in the oxidation state of uranium by the difference in the position of the white line at a resolution better than 1 eV. EXAFS detected changes in the oxygen sublattice as a result of uranium oxidation for the entire series of oxides, which was also previously broadly discussed^20,54,58,59,61,62^.

### Uranium oxides XANES and EXAFS spectra before and after dissolution experiment

According to XANES spectra, the white line positions and the post-edge structures of the spectra are preserved for all oxides before and after exposure to gastrointestinal and lung fluids. This indicates the stability of uranium oxidation state during soaking in liquids of biological significance for a period of time close to the real time the food is in the gastrointestinal tract and for more than one month in the lung fluid (Fig. 4a).

EXAFS spectra of UO_2.05_ before and after exposure in gastrointestinal and lung liquids do not show any significant changes in the local environment of uranium indicative the small influence of gastrointestinal or lungs liquid compositions on oxide behavior (see Fig. 4b, c for experimental and fit EXAFS data and Appendix A9 for fitting parameters).

An acceptable fit of U_4_O_9_ was obtained on the structure solved from neutron diffraction (Appendix A2). At a distance of 2.2–2.8 Å, contributions from three coordination spheres of O atoms are present, with a total CN of 8. Additional coordination spheres with a fixed total CN of 3 appear at a larger distance from U: 3.16 and 3.39 Å. The uranium sublattice in U_4_O_9_ is preserved as in case of perfect fluorite structure and contains 12 U atoms at a distance of 3.89 Å. The U-U distance increases by 0.02 Å in comparison to UO_2_. That can be conditionally significant because of the uncertainty of interatomic distances determination by the EXAFS at the level of ± 0.01 Å.

The Fourier transform of U_4_O_9_ after gastrointestinal liquids (U_4_O_9__G) spectrum is visually well comparable to the spectrum of initial U_4_O_9_. The positions of the maxima of the two main peaks differ within 0.01 Å, while in the case of U_4_O_9__L (after exposure to lung fluid), the first peak is shifted to lower R-values by ~ 0.2 Å. The half-width of the peaks in U_4_O_9__G is larger indicative an increase of the Debye–Waller parameter. Consideration of the real part separately highlights the coincidence of the peaks position corresponding to the contribution of the U-U scattering in all U_4_O_9_ samples.

Visual comparison of the EXAFS spectra of initial U_4_O_9_ and U_4_O_9__G reveals no significant changes in the structure. Therefore, the fitting parameters obtained for untreated U_4_O_9_ are applied to the spectrum of U_4_O_9__G with the following changes, considering the chemistry of the processes and conclusions from the visual comparison of the spectra. ∆E_0_ was fixed to reduce the number of variable parameters, which is motivated by the absence of significant structural changes in the oxide during exposure to gastrointestinal liquids. The approximation for the Debye–Waller parameter of O coordination shells in U_4_O_9__G was taken from untreated U_4_O_9_. The coordination numbers of O coordination spheres are allowed to vary, keeping the overall sum at CN = 11, as it is determined from neutron diffraction of untreated U_4_O_9_.

The resulting fitting model is in line with the expected slightly affected structure of U_4_O_9_. The Debye–Waller parameter for U atoms increases by 0.002 Å^2^, the distance decreases by 0.01 Å. Such a change in the distance, again, is not significant, considering the uncertainty in determining distances by EXAFS of ± 0.01 Å. The parameters of the oxygen spheres change more noticeably: instead of 2 distant subshells in untreated U_4_O_9_ at R > 3 Å, only one remained at 3.38 Å. The distances to the nearest ones initially found at 2.82, 2.46 and 2.26 Å decreased by 0.26, 0.09, and 0.06 Å, respectively. With the length of the U–O bond decrease, the distortions due to treatment with gastrointestinal fluid descends, which can be associated with a stronger energy of short U–O bonds relative to elongated ones.

Thus, treatment of U_4_O_9_ with gastrointestinal fluids does not lead to significant changes in its structure. This process has a significant effect on the oxygen sublattice: the distances to the nearest subshells reduced, and this effect is more pronounced for coordination spheres with long U–O bond lengths. This result is explained by the greater strength of the shortened U–O bonds, which means that they are less susceptible to the exposure in studied liquids (see Fig. 4d, e for experimental and fit EXAFS data and Appendix A9 for fitting parameters).

The spectrum of U_4_O_9_ interacted with lung fluid, on the contrary, shows a significant effect of treatment on the local molecular environment. The real part and the magnitude of the Fourier transform shows significant differences both in the positions and intensities of the main peaks. Due to the visually distinguishable differences between the two spectra, when fitting the EXAFS of the exposed oxide, the structure of the initial U_4_O_9_ is not used as the first approximation. In the U_4_O_9_ oxide exposed to lung fluid a coordination sphere of 2.5 O atoms appears at a much shorter distance of 2.12 Å, which drops out of the range of characteristic values in the initial structure of U_4_O_9_. Such short distances are not described within the framework of the model of formation of cuboctahedral clusters when additional O atoms are included in the fluorite structure. The appearance of a coordination sphere, uncharacteristic for U_4_O_9_, suggests that a rearrangement of the structure upon prolonged interaction with lung fluid occurs. Similar short distances to O atoms, however, are present in the structures of U_4_O_9-y_ defect phases constructed within the model of ordered quad-interstitials clusters^62^. Based on DFT calculations, non-stochiometric U_4_O_9-y_ phases are predicted to be more stable, than U_4_O_9_. In particular, it is shown that U_4_O_8.889_ compound is the most stable in this series^63^. Its structure was taken as the initial approximation when fitting the U_4_O_9_ after lung fluid spectrum. In this model the total CN of all subshells of O atoms, except for the first one, was fixed at 10, so that the sum within the error is close to 11. Splitting of U coordination shell significantly improved the fit. Total CN of two U subshells was constrained to 12. As a result, O coordination shells are located at distances of 2.12, 2.31, 2.54, and 2.76 Å. In the uranium sublattice, at the “fluorite” distance of 3.88 Å, corresponding to the structure of the initial oxide, only a part of U atoms remains with a CN of about 5. The remaining 7 U atoms contribute to the spectrum at a distance of 4.09 Å. The result obtained is in greater agreement with the structure of the nonstoichiometric U_4_O_9-y_ phase than with the structure of the stoichiometric U_4_O_9_. Considering that^63^, it can be assumed that as a result of a long-term treatment of U_4_O_9_ with lung fluid, the structure is reformed into a more stable one, which is not observed in the case of short-term treatment with gastrointestinal fluid.

EXAFS spectra of the initial U_3_O_8_ is fitted according to the structure by Siegel^42^. The best fit is obtained considering only a single coordination shell of equatorial O atoms at 2.23 Å and 2 axial O at 1.98 Å. Although calculated interatomic distances are ~ 0.1 Å shorter than structural ones, the fitting curve agrees well with the data. Elongation of U–O distances and splitting of O_eq._ coordination sphere has resulted in worse fit. Debye–Waller parameters are quite large, which suggests high disordering of O atoms. The best fit of the U coordination sphere is obtained with 3 U subshells according to the layered structure of U_3_O_8_: 2 and 4 atoms at 3.70 and 3.91 Å, respectively, represent neighboring U atoms within a layer, 2 U atoms at 4.20 Å are U of the adjacent sheets. The obtained fitting model agrees well both with data and known structural information.

The comparison of a real parts of Fourier transformed EXAFS spectra of U_3_O_8_ samples before and after treatment clearly indicates the ordering of O subshells after gastrointestinal liquids. Inconsistencies at 3.5–4 Å suggest that some changes occur in the nearest U subshell that contains the contribution of in-layer U atoms. The overall shape of the EXAFS spectra indicates that no significant changes in the structure happens after the exposure of U_3_O_8_ to gastrointestinal and lung liquids. Therefore, EXAFS model obtained for untreated U_3_O_8_ is applied to samples of treated U_3_O_8_. In U_3_O_8__L, O coordination spheres appeared at 0.03–0.05 Å shorter distances and with lower Debye–Waller factors, verifying the suggestion drawn from the shape of FT real part spectra. Distance to the first U subshell increases by 0.03–0.05 Å, while Debye–Waller factors remains the same (see Fig. 4f, g for experimental and fit EXAFS data and Appendix A9 for fitting parameters).

According to the EXAFS analysis, soaking process leads to ordering of U–O subshells, decrease of U–O distances and slightly affects U sublattice in U_3_O_8_. That is consistent with the suggestion about higher resistance of U_3_O_8_ under oxidizing conditions^64^.

Fourier transforms of α-UO_3_ samples also look similarly, except of the sample treated with lung fluid, which demonstrates higher background at low R. The untreated sample of α-UO_3_ is fitted considering layered structure with 2 axial O atoms at shorter distance and 6 O atoms in equatorial plane at 2.2–2.8 Å. CN for the axial O subshell is kept fixed equal to 2. For equatorial O CN is allowed to vary so that the sum remains equal to 6. Split of the equatorial subshell is allowed only if it led to the improvement of the fit quality. Following this approach, 3 coordination subshells are introduced in the final fit: at 2.28, 2.56 and 2.76 Å. CNs for U-U are kept constant according to the structure and obtained R and σ^2^ are reasonable. The similarity of spectra in R-space indicates the absence of significant structural changes after soaking in gastrointestinal and lung liquids. Therefore, fitting model obtained for α-UO_3_ is used as the first approximation in the fitting procedure of the spectra of treated samples. Calculated parameters demonstrate the absence of fundamental structural changes after the exposure of UO_3_ to gastrointestinal and lung liquids, with only minor changes in CN ratios in O_eq._ subshells (see Fig. 4h, i for experimental and fit EXAFS data and Appendix A9 for fitting parameters).

Features at low R around 1 Å are not described by EXAFS equation and are attributed to background or truncation effects. They don't have any physical sense and therefore were not considered in the fitting procedure.

The obtained data confirms the stability of α-UO_3_ with respect to the treatment with human body liquids, which is not the case of the other oxides, i.e. U_4_O_9_, U_3_O_8_ and UO_2.05_. According to the EXAFS fitting results, in U_3_O_8_ and UO_2.05_ the subsequent ordering of O sublattice took place. The most prominent changes are observed in U_4_O_9_ exposed to lung liquid for 34 days: the initial structure undergo transformations and form different, but more stable structure presumably of non-stochiometric U_4_O_9-y_ phase. Significant lattice ordering due to fluid exposure occurred only for U_4_O_9_ in the simulated lung fluid, while the lattice parameters changed for other oxides only for 0.01–0.02 angstroms. During oxidation in a series UO_2+x_ a decrease in the interatomic distance occurs with an increase in the saturation of the lattice with oxygen^58^*.*

## Conclusion

For characterization of uranium oxides transformation in gastrointestinal and lung fluids, the series of uranium oxides are synthetized and characterized by XAS and Raman spectroscopy methods. Exposure of uranium oxides to lung fluid caused more dramatic transformation of solid-state oxides surface, according to Raman spectroscopy, than to gastrointestinal fluids due to longer duration of the experiment. Continuance of interaction of uranium oxides with liquids showed up to have more influence on oxide transformation than lower pH in gastrointestinal fluids. The structure of α-UO_3_ oxide remained insignificantly changed after the exposure to human body liquids, which is not the case of U_4_O_9_, U_3_O_8_ and UO_2.05_. Oxygen sublattice of U_3_O_8_ and UO_2.05_ after soaking in liquids became more ordered than in the initial oxides. The most significant changes took place in U_4_O_9_ after exposure to lung liquid. According to EXAFS results, its initial structure undergoes transformations and form different, but more stable structure of non-stochiometric U_4_O_9−y_ phase. It contradicts with the fact that the most soluble uranium is in oxidation state + 6. Further research should include U_4_O_9-y_ stability and properties studies.

## Supplementary Information

Supplementary Information.

## Data Availability

The datasets generated and analyzed during the current study are not publicly available since the given spectral data are sufficient to understand the operation but they are available from the corresponding author.
