# Supplementary material for: Uranium oxides structural transformation in human body liquids
Source: Sci Rep. 2023 Mar 11;13:4088. doi: 10.1038/s41598-023-31059-z (PMC10008576; doi:10.1038/s41598-023-31059-z)
Supplement: Supplementary file 1 — Supplementary Information. [file 41598_2023_31059_MOESM1_ESM.pdf]

Uranium oxides structural transformation in human body liquids

Tatiana Poliakova[1], Anna Krot[1], Alexander Trigub[2], Iurii Nevolin[3], Alexey Averin[3], Vasiliy Yapaskurt[4], Irina Vlasova[1], Petr Matveev[1] and Stepan Kalmykov[1,3,*]

Appendix

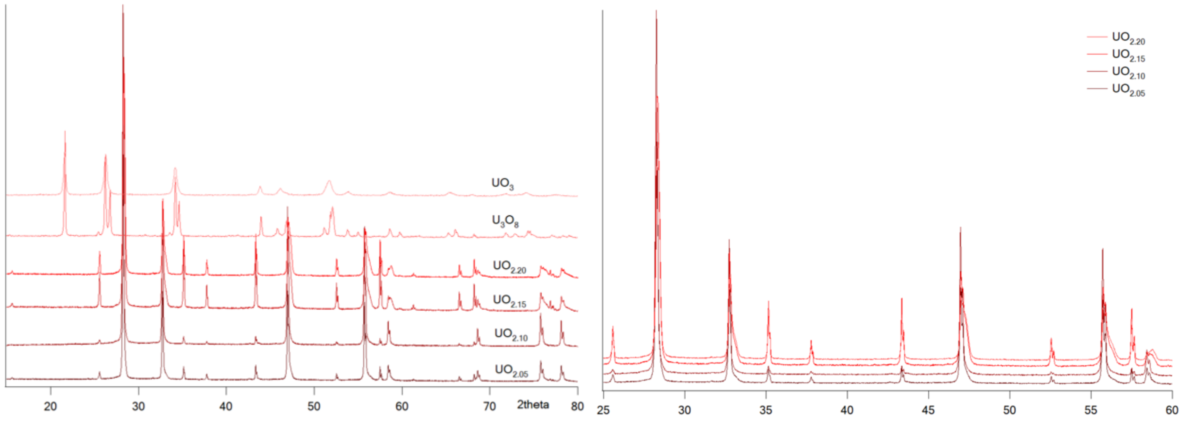

A1. X-ray diffraction of synthetized oxides

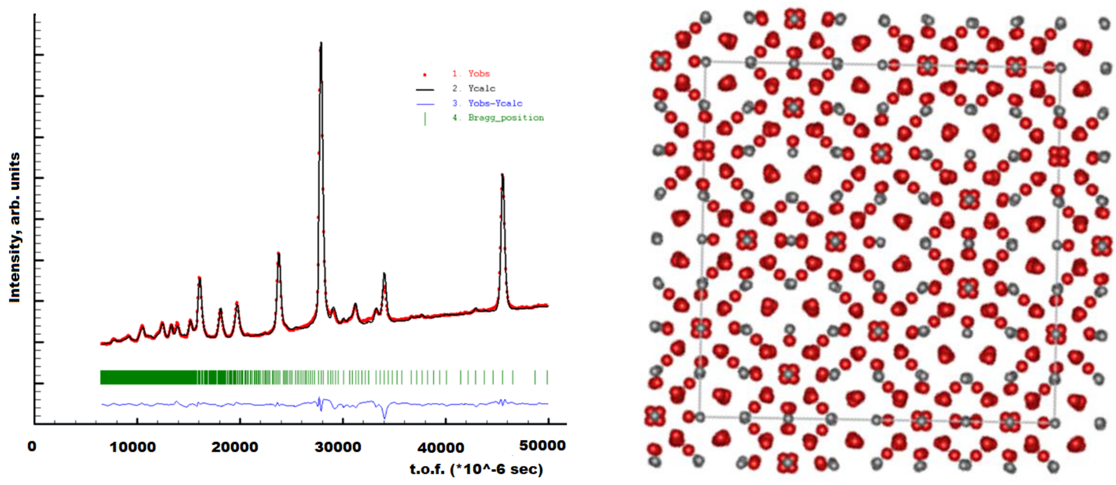

A2. Neutron diffraction and crystal cell of U_4O_9

a, Å	21.78530	δa	0.00081
b, Å	21.78530	δb	0.00081
c, Å	21.78530	δc	0.00081
α, deg	90.00000	δα	0.00000
β, deg	90.00000	δβ	0.00000
γ, deg	90.00000	δγ	0.00000

A3. Cell parameters of U_4O_9

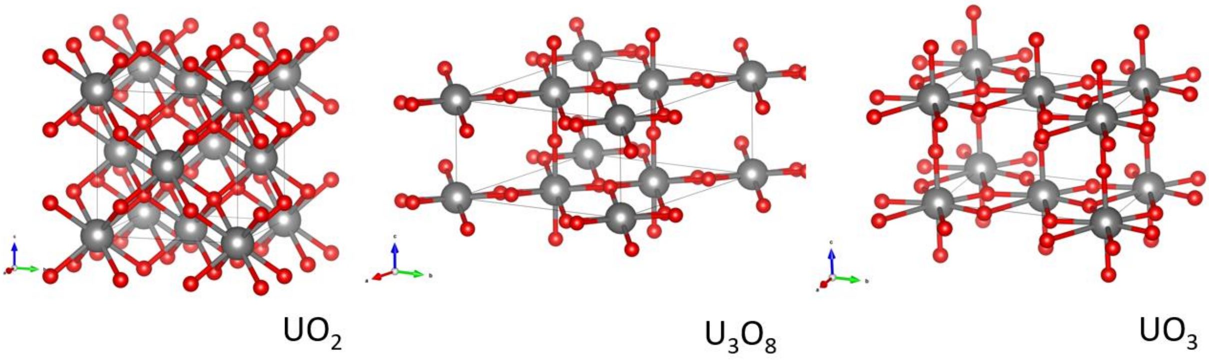

A4. Crystal cells of uranium oxides

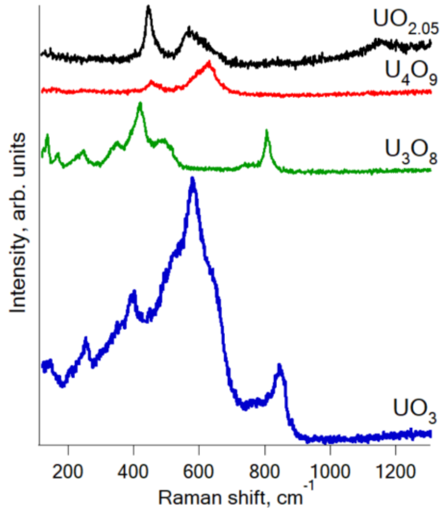

A5. Raman spectra of synthetized $UO_{2.05}$, U_4O_9, U_3O_8 and UO_3 before dissolution

Oxide	U_3O_8		U_3O_8 – G		U_3O_8 – L	
Peak, cm^{-1}	Height	FWHM	Height	FWHM	Height	FWHM
347 A_{1g} U-O	171	48	120	44	135	35
418 A_{1g} U-O	392	48	290	50	301	47
454 T_{2g} U-O	-		89	13	-	
475 E_g U-O	172	33	221	32	48	14
505 E_g U-O	170	45	234	29	140	48

A6. Deconvolution of Raman spectra of U_3O_8

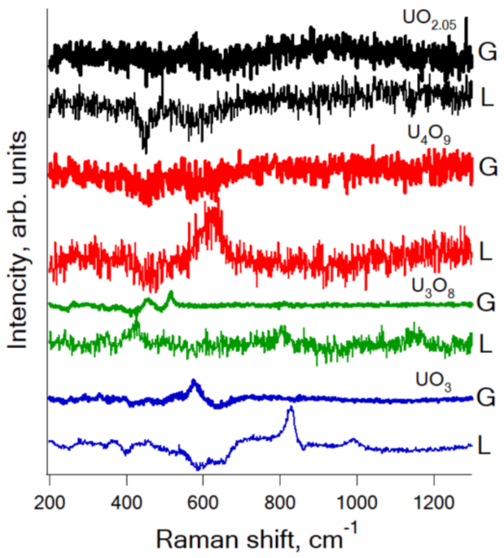

A7. Residual of Raman spectra of synthetized $UO_{2.05}$, U_4O_9, U_3O_8 and UO_3 before and after soaking in gastrointestinal and lung fluids.

Mode, cm^{-1}	Comment	Oxide	References
160	B_{1g} vibration	α-U_3O_8	[26]
230	More intensive with oxidation state increase	UO_2, UO_{2+x}, α-U_3O_8, γ-UO_3	[22,23,26]
323/335	Valent vibration U-O A_{1g}	α-U_3O_8	[22]
445	Three-fold degenerate Raman-active oscillation T_{2g}	UO_2, UO_{2+x}, U_4O_9, U_3O_7	[16,17,22,23,26,42,43,45]
560/585	Longitudinal optical phonon of the first order LO	UO_2	[13,27,43,45]
630/640	Distortion of the anionic sublattice in the structure of fluorite due to the incorporation of oxygen	UO_2, $UO_{2,11}$, $UO_{2,15}$, $UO_{2,17}$, $UO_{2,20}$, $UO_{2,24}$, U_4O_9	[27,45]
730/750	Two valent vibrations U-O A_{2u}	α-U_3O_8	[17,26,43]
800/810	Contribution of octahedral UO_3 to α-U_3O_8 or uranyl symmetrical axial extension	α-U_3O_8	[17,26,42,43]
1150	Longitudinal optical phonon of the second order 2LO	UO_2, UO_{2+x}	[22,26,27,42,43]

A8. Combination scattering modes in uranium oxides

Sample	ΔE_0, eV	U-O			U-U		
		CN	R, Å	σ^2, Å^2	CN	R, Å	σ^2, Å^2
$UO_{2.05}$	3.3	3.1	2.24	0.005	**12**	3.87	0.005
		5.5	2.39				
$UO_{2.05}$_L	4.4	0.2	2.23	0.010	**12**	3.87	0.005
		7.8	2.35				
$UO_{2.05}$_G	4.4	0.4	1.73	0.007	**12**	3.87	0.006

		5.9	2.34				
		1.6	2.86				
$UO_{2.10}$	3.2	8.2	2.35	0.010	**12**	3.87	0.005
		1.0	2.81				
$UO_{2.15}$	2.9	8.5	2.34	0.015	**12**	3.87	0.008
		1.3	2.75				
$UO_{2.20}$	3.7	8.7	2.34	0.017	**12**	3.88	0.008
		1.7	2.74				
U_4O_9	4.1	**4**	2.26	0.008	**12**	3.89	0.011
		**3**	2.46				
		**1**	2.82				
		1.0	3.17	0.003			
		2.0	3.40				
U_4O_9_L	-8.0	2.5	2.12	0.003	4.9	3.88	0.009
		3.2	2.31	0.003	7.1	4.09	
		2.2	2.54				
		3.6	2.76				
U_4O_9_G	**3.9**	2.8	2.20	0.007	**12**	3.88	0.012
		4.2	2.37				
		2.0	2.56				
		2.0	3.38				
U_3O_8	3.4	**2**	1.98	0.014	**2**	3.70	0.006
		**5**	2.22		**4**	3.91	
					**2**	4.22	
U_3O_8_L	**3.4**	**2**	1.94	0.012	**2**	3.73	0.006
		**5**	2.19		**4**	3.90	
					**2**	4.22	
U_3O_8_G	**3.4**	**2**	1.94	0.010	**2**	3.75	0.006
		**5**	2.19		**4**	3.93	
					**2**	4.22	
UO_3	-8.0	**2**	2.13	0.003	**2**	3.94	
		1.1	2.28	0.003	**4**	4.14	0.007
		2.4	2.56		**2**	4.47	
		2.5	2.76				
UO_3_G	-7.7	**2**	2.17	0.003	**2**	3.95	0.006
		1.3	2.38	0.003	**4**	4.14	
		2.5	2.60		**2**	4.47	
		2.2	2.78				
UO_3_L	-7.7	**2**	2.13	0.003	**2**	3.96	
		2.0	2.28	0.004	**4**	4.15	0.008
		1.9	2.56		**2**	4.48	
		2.1	2.76				

A9. EXAFS fitting parameters bold font indicates that the parameter was fixed during fitting.
